# Clinical Outcome of Eosinophilic Airway Inflammation in Chronic Airway Diseases Including Nonasthmatic Eosinophilic Bronchitis

**DOI:** 10.1038/s41598-017-18265-2

**Published:** 2018-01-09

**Authors:** Jaeyoung Cho, Sun Mi Choi, Jinwoo Lee, Young Sik Park, Sang-Min Lee, Chul-Gyu Yoo, Young Whan Kim, Sung Koo Han, Chang-Hoon Lee

**Affiliations:** 1 Division of Pulmonary and Critical Care Medicine, Department of Internal Medicine, Seoul National University Hospital, Seoul, Republic of Korea; 2 Department of Internal Medicine, Seoul National University College of Medicine, Seoul, Republic of Korea

## Abstract

We enrolled patients with confirmed sputum eosinophilia who had visited our tertiary referral hospital between 2012 and 2015. We evaluated the incidence and predictors of exacerbations in patients with nonasthmatic eosinophilic bronchitis (NAEB), and investigated predictors of improvement in eosinophilic inflammation in chronic airway diseases with or without persistent airflow limitation. In total, 398 patients with sputum eosinophilia were enrolled. Of these, 152 (38.2%) had NAEB. The incidence rate of exacerbations requiring treatment with antibiotics, systemic corticosteroids, or hospital admission was 0.13 per patient-year (95% CI, 0.06–0.19) in NAEB. Inhaled corticosteroid (ICS) did not affect the risk of exacerbations, even in an analysis of propensity score. One hundred seventy-six patients had chronic airway diseases; in 37 of these (21.0%), sputum eosinophilia had improved at the 1-year follow-up. Patients who had persistent airflow limitation were less likely to show an improvement in eosinophilic inflammation (aOR, 0.26; 95% CI, 0.09–0.77) when they were treated with ICSs for less than 75% of the follow-up days. Exacerbations requiring systemic corticosteroids, antibiotics, or hospitalization did occur, although infrequently, in NAEB patients. Among patients with chronic airway diseases, those with persistent airflow limitation were less likely to show an improvement in eosinophilic airway inflammation.

## Introduction

Nonasthmatic eosinophilic bronchitis (NAEB) usually presents with corticosteroid-responsive chronic cough; in fact, the condition is diagnosed in 13% to 33% of patients with chronic cough referred for specialist opinion^1–4^. Patients with NAEB have eosinophilic airway inflammation, which manifests as sputum eosinophilia similar to that in asthma. However, these patients lack evidence of variable airflow limitation or airway hyperresponsiveness. Previous longitudinal studies on NAEB have focused on the prognosis concerning relapse and the development of chronic airflow obstruction^5–7^. However, NAEB patients could have respiratory symptoms other than cough, such as chest tightness with wheezing, shortness of breath, and sputum production^8–10^, and treatment with systemic corticosteroids is occasionally required to relieve these symptoms^11^. No studies have yet investigated the incidence and predictors of acute exacerbations, defined as worsened respiratory symptoms requiring systemic treatment, in this condition.

Sputum eosinophilia is also present in 38% to 44% of patients with persistent airflow limitation, including those with chronic obstructive pulmonary disease (COPD)^12,13^. Patients with COPD who show eosinophilic airway inflammation respond better to inhaled corticosteroids (ICSs)^12,14^ and systemic corticosteroids^13,15^, as do those with asthma-COPD overlap syndrome (ACOS)^16^. However, it is not yet clear whether the outcome of eosinophilic airway inflammation differs depending on whether persistent airflow limitation—a characteristic of COPD—is present.

Our study aimed to investigate the incidence and predictors of exacerbations in NAEB patients, and to identify predictors of improvement in sputum eosinophilia in chronic airway diseases, including NAEB, asthma, and COPD.

## Materials and Methods

### Patients

Our retrospective cohort study included patients with sputum eosinophilia (≥3%) who had presented at Seoul National University Hospital between March 2012 and June 2015. Patients were excluded according to the following criteria: (1) no initial pulmonary function tests were conducted within 3 months of the initial induced sputum tests; (2) they had active pulmonary tuberculosis or destroyed lung by tuberculosis (parenchymal damage to more than one lung lobe); (3) they had bronchiectasis (more than one lung lobe); or (4) they had eosinophilic pneumonia.

NAEB was diagnosed using the following criteria: (1) prolonged (>8 weeks) respiratory symptoms, including cough; (2) no abnormality on chest radiograph; (3) postbronchodilator forced expiratory volume in 1 second (FEV_1_)/forced vital capacity (FVC) ≥70% predicted; (4) negative response to a short-acting bronchodilator, and absence of airway hyperresponsiveness to inhaled methacholine or mannitol; and (5) sputum eosinophilia (≥3%). Asthma was diagnosed in cases of positive bronchodilator response or airway hyperresponsiveness according to the Global Initiative for Asthma 2016^17^, whereas COPD was identified in cases of postbronchodilator FEV_1_/FVC <70% predicted. When patients fulfilled the diagnostic criteria for both asthma and COPD, we defined their condition as possible ACOS. Both NAEB and diseases with chronic airflow obstruction (asthma, COPD, and possible ACOS) were defined as chronic airway diseases.

At the initial visits, patients with chronic respiratory symptoms were examined using induced sputum tests, chest radiograph, pulmonary function tests with bronchodilator responses, and bronchial provocation tests. About half of the study patients were followed up with induced sputum tests for at least 1 year. The present study was approved by the institutional review board of the Seoul National University Hospital (H-1602-126-743) and was conducted in accordance with the Declaration of Helsinki. The requirement for informed consent was waived.

### Measurement

The following clinical data were collected for analysis: age, sex, smoking status, and baseline symptom scores (cough score, COPD assessment test score^18^, and asthma control test score^19^). The cough score was assessed using a numeric rating scale ranging from 0 to 5 (0 = no cough, 5 = cough all the time). Adherence to ICSs was assessed using the medication possession ratio (MPR), which estimates the percentage of days’ supply obtained during the follow-up period.

A positive bronchodilator response was defined as an increase in FEV_1_ of ≥12% and ≥200 mL from baseline after inhalation of 200 µg of salbutamol. Airway hyperresponsiveness was identified using bronchial provocation tests; these were considered positive if the FEV_1_ fell by ≥20% after a methacholine dose of <16 mg/mL^20^, or if the FEV_1_ had fallen by ≥15% before the last dose of mannitol (before a cumulative mannitol dose of 635 mg had been administered)^21^.

The patients’ atopic status was determined using either skin prick testing to 55 common inhalant allergens or immunoglobulin E (IgE) specific to *Dermatophagoides pteronyssinus* and *Dermatophagoides farinae* measured by an ImmunoCAP 250 analyzer (ThermoFisher, Uppsala, Sweden). Specific IgE sensitization was dichotomized at a cut-off of 3.50 kU/L. Atopy was defined as either a positive skin prick test or a positive specific IgE measurement to above house dust mites.

Induced sputum tests were conducted as previously described^22^. Briefly, sputum was induced using a 4.5% hypertonic saline inhalation, administered through an ultrasonic nebulizer (Omron; Tokyo, Japan) for 5–20 min, with the output set at 4.5 mL/min. The sputum was mixed with an equal volume of 0.01 M dithioerythritol, filtered using a 100-µm cell strainer or mesh on a 15-mL tube, and centrifuged at 400–800 g for 10 min at 4 °C. The cell pellets were resuspended using phosphate-buffered saline. Cytospins were performed at 42 g for 5 min; the cells then underwent Diff-Quik staining. Differential cell counts were expressed as percentages of 300 non-squamous cells.

### Data Analysis

Clinical characteristics were compared between groups using either the independent samples *t*-test or one-way analysis of variance for continuous variables. For categorical variables, they were compared using either the χ^2^ test or Fisher’s exact test.

Data analyses were carried out independently in two subcohorts. First, in a subcohort of patients who had NAEB diagnosed at baseline (subcohort 1), the incidence rate of moderate or severe exacerbations was estimated, and the incidence rate ratio was calculated—using a negative binomial regression model—between the group with an MPR for ICSs of <50% and that with an MPR for ICSs of ≥50%. An exacerbation was defined as any worsening of respiratory symptoms that led to treatment with systemic corticosteroids, antibiotics, or both (moderate), to hospital admission, or to emergency department visits (severe). This definition is widely applied in studies involving smokers^23^ and chronic respiratory diseases (asthma, COPD, and destroyed lung by tuberculosis^24^). The time to the first moderate or severe exacerbation was analyzed using the Kaplan–Meier method after propensity score (PS) matching, which provided 33 pairs of patients, each pair comprising one with an MPR for ICSs of <50% and the other with an MPR for ICSs of ≥50%. We also estimated the incidence rate of the development of chronic airflow obstruction in subcohort 1. Second, in a subcohort of patients with sputum eosinophilia who had been followed up with induced sputum tests for at least 1 year (subcohort 2), we used a logistic regression model to identify predictors of improvement in sputum eosinophilia. Improvement in sputum eosinophilia was defined as a decrease in induced sputum eosinophil count to <3%. The multivariable analysis was adjusted for persistent and variable airflow limitation, and variables with *P* values < 0.1 in univariable analyses considering collinearity. Persistent airflow limitation was identified in cases of postbronchodilator FEV_1_/FVC < 70% predicted, and variable airflow limitation was defined in cases of positive bronchodilator response or airway hyperresponsiveness. The 1-year change in sputum eosinophils was analyzed using a random-slope linear mixed model after PS matching, which provided 47 pairs of patients, each pair comprising one with an MPR for ICSs of <75%, and the other with an MPR for ICSs of ≥75%.

*P* values less than 0.05 were considered significant. Statistical analyses were performed using Stata statistical software (Version 14.2; StataCorp LP, College Station, TX).

### Data Availability

The datasets generated during and/or analyzed during the current study are available from the corresponding author on reasonable request.

## Results

### Patient Characteristics

**Figure 1:**
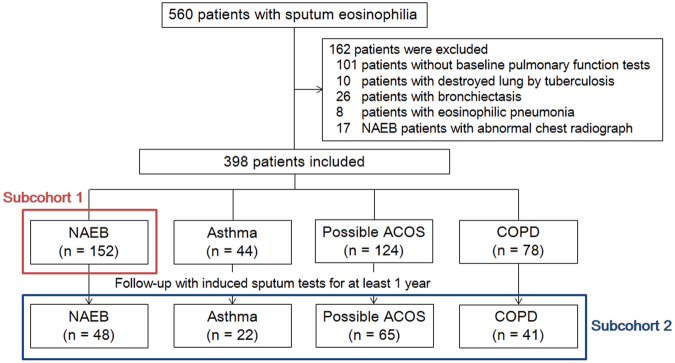
Flowchart of patient selection. Subcohort 1 was composed of all patients diagnosed with nonasthmatic eosinophilic bronchitis. Subcohort 2 was composed of patients with sputum eosinophilia who were followed up with induced sputum tests for at least 1 year. When patients fulfilled both diagnostic criteria for asthma and COPD, we defined their condition as possible ACOS. ACOS, asthma-COPD overlap syndrome; COPD, chronic obstructive pulmonary disease; NAEB, nonasthmatic eosinophilic bronchitis.

A total of 560 patients with sputum eosinophilia were enrolled. Of these, 162 were excluded from the study (Fig. 1). Of the resulting 398 patients, 152 had NAEB (subcohort 1); Table 1 summarizes their clinical characteristics. Only one fourth of the NAEB patients had received ICS therapy for ≥50% of the follow-up days, and less than one sixth had received it for ≥75% of the follow-up days.

**Table 1:** Clinical Characteristics of 152 Patients With Nonasthmatic Eosinophilic Bronchitis.

Characteristic	N = 152
Age, y	58.9 ± 13.8
Female sex	109 (71.7)
Smoking status	
Never-smoker	106 (69.7)
Former smoker	18 (11.8)
Current smoker	8 (5.3)
Unknown	20 (13.2)
Baseline symptom scores (n = 103)	
Cough score (n = 75)	2.0 ± 1.4
CAT score (n = 76)	12.8 ± 6.5
ACT score (n = 100)	19.4 ± 4.5
White blood cell,/μL (n = 96)	6151 ± 1776
Blood eosinophil, % (n = 96)	3.0 ± 2.2
Blood eosinophil ≥5%	16 (16.7)
Blood eosinophil ≥3%	37 (38.5)
Blood eosinophil,/μL (n = 96)	187 ± 156
Blood eosinophil ≥500/μL	5 (5.2)
Serum IgE, U/mL (n = 43)	200 ± 676
Serum IgE ≥100 U/mL	13 (30.2)
Positive skin prick test (n = 103)^a,b^	22 (21.4)
Positive specific IgE to house dust mite (n = 30)^a^	1 (3.3)
Positive to *D*. *pteronyssinus*	1 (3.3)
Positive to *D*. *farinae*	1 (3.3)
Postbronchodilator FEV_1_, % predicted	108.7 ± 17.2
Postbronchodilator FEV_1_/FVC, %	80.0 ± 5.7
Bronchodilator response (FEV_1_, %)	3.1 ± 3.5
Bronchodilator response (FEV_1_, mL)	70.8 ± 80.4
Sputum eosinophil, %	8.7 ± 9.0
Sputum neutrophil, %	1.7 ± 2.4
Use of ICS	74 (48.7)
MPR for ICS, %	25.5 ± 33.0
MPR for ICS ≥75%	23 (15.1)
MPR for ICS ≥50%	36 (25.7)

Data are presented as mean ± SD or No. (%).

Abbreviations: ACT, asthma control test; CAT, COPD assessment test; FEV_1_, forced expiratory volume in 1 second; FVC, forced vital capacity; ICS, inhaled corticosteroid; MPR, medication possession ratio.

^a^Of 152 NAEB patients in subcohort 1, 123 (80.9%) underwent either skin prick testing to 55 common inhalant allergens or measurement of the specific IgE to house dust mites (*Dermatophagoides pteronyssinus* and *Dermatophagoides farinae*). Of the 123 patients, 93 underwent only skin prick testing, 20 underwent only measurement of the specific IgE, and 10 underwent both.

^b^Of 103 NAEB patients who underwent skin prick testing to 55 common inhalant allergens, 13 showed the positive test to *D*.*pteronyssinus*, 14 showed the positive test to *D*.*farinae*.

### Exacerbations in NAEB patients

**Figure 2:**
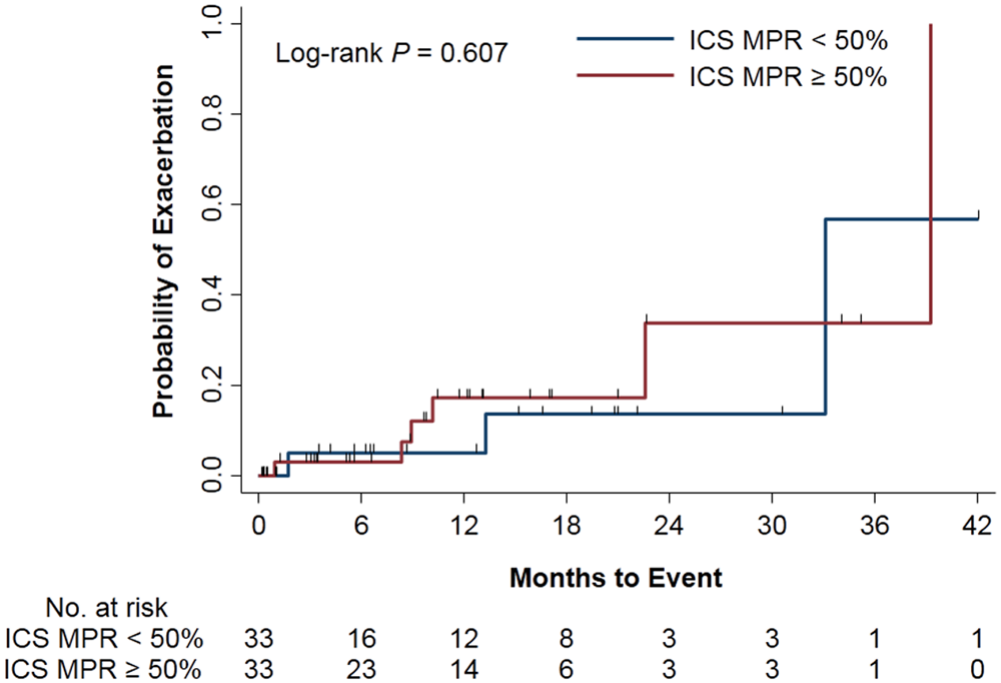
Kaplan–Meier curves of the time to first moderate or severe exacerbation in patients with nonasthmatic eosinophilic bronchitis (the propensity score–matched cohort). ICS, inhaled corticosteroid; MPR, medication possession ratio.

Of the 152 NAEB patients, 15 (9.9%) experienced 16 moderate or severe exacerbations during the follow-up period (median, 6.4 months; interquartile range [IQR], 0.7–15.3 months). The incidence rate of moderate or severe exacerbations was 0.13 per patient-year (95% CI, 0.06–0.19 per patient-year). To investigate whether ICSs affect exacerbations, PS matching was applied (MPR for ICSs, <50% vs ≥50%). The clinical characteristics of subcohort 1 were comparable after PS matching (S1 Table). The incidence rates of exacerbations in each group were not significantly different in the PS matched-cohort (incidence rate ratio, 1.72; 95% CI, 0.44–6.63; Table 2). Furthermore, the time to the first moderate or severe exacerbation did not differ significantly between the groups (log-rank *P* = 0.607; Fig. 2).

**Table 2:** Incidence Rates of Moderate or Severe Exacerbations in Patients With Nonasthmatic Eosinophilic Bronchitis.

Variable	Before Propensity Score Matching					After Propensity Score Matching				
	MPR for ICSs <50% (n = 113)		MPR for ICSs ≥50% (n = 39)		IRR (95% CI)	MPR for ICSs <50% (n = 33)		MPR for ICSs ≥50% (n = 33)		IRR (95% CI)
	n	IR	n	IR		n	IR	n	IR	
Exacerbation	8	0.10	8	0.18	1.87 (0.70–5.00)	3	0.11	7	0.19	1.72 (0.44–6.63)

Abbreviations: CI, confidence interval; ICS, inhaled corticosteroid; IR, incidence rate (per patient-year); IRR, incidence rate ratio; MPR, medication possession ratio.

### Chronic Airflow Obstruction in NAEB Patients

Of the 48 NAEB patients who were followed-up with induced sputum tests for at least 1 year, 46 underwent additional yearly pulmonary function tests with bronchodilator response or provocation tests during the follow-up period (median, 16.2 months; IQR, 12.7–25.1 months). Among them, chronic airflow obstruction developed in five patients (asthma: three patients, COPD: two patients). The incidence rate of the development of chronic airflow obstruction was 0.07 per patient-year (95% CI, 0.03–0.16 per patient-year). The median time to the development of chronic airflow obstruction was 14.2 months (IQR, 12.5–25.1 months).

### Characteristics and Outcomes of Patients With Sputum Eosinophilia

The clinical characteristics of subcohort 2 are summarized in Table 3. About 4% of NAEB patients experienced moderate or severe exacerbations during the 1-year follow-up period, whereas about 27% of those with asthma and possible ACOS, and 12% of those with COPD did. Of the 176 patients in subcohort 2, 37 (21.0%) showed an improvement in sputum eosinophilia at the 1-year follow-up. Patients with NAEB were more likely to show an improvement in sputum eosinophilia than those with other chronic airway disease (NAEB: 35.4%, asthma: 18.2%, possible ACOS: 16.9%, and COPD: 12.2%; *P* = 0.044; Table 3).

**Table 3:** Clinical Characteristics and Outcomes in 176 Patients With Sputum Eosinophilia.

Characteristic	NAEB (n = 48)	Asthma (n = 22)	Possible ACOS^a^ (n = 65)	COPD (n = 41)	*P* Value
Age, y	61.7 ± 13.8	56.9 ± 13.9	68.8 ± 8.8	69.0 ± 7.2	<0.001
Female sex	34 (70.8)	11 (50.0)	13 (20.0)	8 (19.5)	<0.001
Smoking status					<0.001
Never-smoker	36 (75.0)	13 (59.1)	11 (16.9)	11 (26.8)	
Former smoker	8 (16.7)	7 (31.8)	37 (56.9)	20 (48.8)	
Current smoker	2 (4.2)	1 (4.6)	16 (24.6)	10 (24.4)	
Unknown	2 (4.2)	1 (4.6)	1 (1.5)	0	
Baseline symptom scores (n = 150)					
Cough score (n = 98)	1.9 ± 1.2	2.0 ± 1.0	1.8 ± 1.4	1.9 ± 1.5	0.981
CAT score (n = 123)	12.6 ± 7.1	17.7 ± 9.8	12.9 ± 7.9	13.7 ± 8.0	0.716
ACT score (n = 137)	20.6 ± 4.4	16.4 ± 6.6	19.1 ± 4.5	18.9 ± 4.6	0.035
White blood cell,/μL (n = 119)	6211 ± 1543	7651 ± 1685	6982 ± 2242	7313 ± 2571	0.112
Blood eosinophil, % (n = 119)	3.2 ± 2.6	5.0 ± 4.3	3.8 ± 3.0	2.3 ± 1.1	0.020
Blood eosinophil ≥5%	3 (10.3)	7 (43.8)	14 (29.2)	0	<0.001
Blood eosinophil ≥3%	11 (37.9)	9 (56.3)	26 (54.2)	8 (30.8)	0.161
Blood eosinophil,/μL (n = 119)	201.1 ± 175.6	391.6 ± 354.6	262.7 ± 214.5	156.8 ± 66.4	0.004
Blood eosinophil ≥500/μL	3 (10.3)	3 (18.8)	6 (12.5)	0	0.142
Serum IgE, U/mL (n = 68)	72.2 ± 79.2	269.5 ± 545.2	281.7 ± 565.6	87.8 ± 83.7	0.353
Serum IgE ≥100 U/m	4 (30.8)	3 (37.5)	14 (42.4)	3 (21.4)	0.608
Positive skin prick test (n = 121)^b^	3 (10.0)	5 (38.5)	9 (17.0)	4 (16.0)	0.190
Positive specific IgE to house dust mite (n = 62)^b^	0	2 (25.0)	2 (6.3)	0	0.153
Postbronchodilator FEV_1_, % predicted	109.3 ± 19.8	100.4 ± 17.5	80.9 ± 15.3	86.4 ± 24.7	<0.001
Postbronchodilator FEV_1_/FVC, %	79.3 ± 5.1	78.3 ± 5.8	54.3 ± 9.0	58.7 ± 11.1	<0.001
Bronchodilator response (FEV_1_, %)	3.6 ± 3.8	7.2 ± 5.7	11.9 ± 11.5	4.9 ± 4.7	<0.001
Bronchodilator response (FEV_1_, mL)	81.3 ± 84.5	151.4 ± 130.9	195.5 ± 176.2	79.3 ± 79.2	<0.001
Sputum eosinophil, %	9.0 ± 9.5	13.7 ± 10.3	12.9 ± 8.2	12.8 ± 10.6	0.098
Sputum neutrophil, %	1.1 ± 0.9	3.2 ± 3.7	3.1 ± 3.8	3.4 ± 11.2	0.229
Use of ICS during the 1-year follow-up	35 (72.9)	20 (90.9)	44 (67.7)	26 (63.4)	0.121
MPR for ICS during the 1-year follow-up, %	41.3 ± 34.7	69.7 ± 39.1	50.0 ± 41.1	39.9 ± 38.6	0.018
MPR for ICS ≥75%	10 (20.8)	12 (54.6)	26 (40.0)	8 (19.5)	0.005
MPR for ICS ≥50%	23 (47.9)	16 (72.7)	36 (55.4)	17 (41.5)	0.101
Patients with moderate or severe exacerbations during the first year	2 (4.2)	6 (27.3)	18 (27.7)	5 (12.2)	0.003
Improvement in sputum eosinophilia (<3%) after 1 year	17 (35.4)	4 (18.2)	11 (16.9)	5 (12.2)	0.044

Data are given as mean ± SD or No. (%).

Abbreviations: ACOS, asthma-COPD overlap syndrome; ACT, asthma control test; CAT, COPD assessment test; COPD, chronic obstructive pulmonary disease; FEV_1_, forced expiratory volume in 1 second; FVC, forced vital capacity; ICS, inhaled corticosteroid; MPR, medication possession ratio; NAEB, nonasthmatic eosinophilic bronchitis.

^a^When patients fulfilled both diagnostic criteria for asthma and COPD, we defined their condition as possible ACOS.

^b^Of 176 patients in subcohort 2, 146 (83.0%) underwent either skin prick testing to 55 common inhalant allergens or measurement of the specific IgE to house dust mites (*Dermatophagoides pteronyssinus* and *Dermatophagoides farinae*). Of the 146 patients, 84 underwent only skin prick testing, 25 underwent only measurement of the specific IgE, and 37 underwent both.

### Predictors of Improvement in Sputum Eosinophilia

Table 4 shows the univariable analysis of factors associated with a 1-year improvement in sputum eosinophilia. Because there was a statistically significant interaction between persistent airflow limitation and MPR for ICSs (*P* for interaction = 0.027), the multivariable analysis was performed in each subgroup stratified in terms of MPR for ICSs (<75% vs ≥75%). After adjustment for blood eosinophils, serum IgE, FEV_1_% predicted, sputum eosinophils, and variable airflow limitation, patients who had persistent airflow limitation and had received ICS therapy for less than 75% of the follow-up days were less likely to show an improvement in eosinophilic airway inflammation (aOR, 0.26; 95% CI, 0.09–0.77; *P* = 0.015; Table 5). After PS matching for the 1-year MPR for ICSs (<75% vs ≥75%; S2 Table), ICS use had no significant impact on the decrease in sputum eosinophils in the linear mixed model (*P* for interaction = 0.101).

**Table 4:** Univariable Analysis of Factors Associated With 1-year Improvement in Sputum Eosinophilia.

Characteristic	No Improvement (n = 139)	Improvement (n = 37)	*P* Value
Age, y	66.2 ± 11.1	62.7 ± 13.0	0.104
Female sex	50 (36.0)	16 (43.2)	0.417
Smoking			0.760
Never-smoker	54 (38.9)	17 (46.0)	
Former smoker	59 (42.5)	13 (35.1)	
Current smoker	23 (16.6)	6 16.2)	
Unknown	3 (2.2)	1 (2.7)	
Baseline symptom scores (n = 150)			
Cough score (n = 98)	2.0 ± 1.4	1.5 ± 1.3	0.108
CAT score (n = 123)	14.2 ± 7.6	9.1 ± 7.3	0.004
ACT score (n = 137)	18.7 ± 5.0	20.3 ± 4.8	0.138
White blood cell,/μL	7069 ± 2302	6532 ± 1279	0.128
Blood eosinophil, % (n = 119)	3.8 ± 3.1	2.6 ± 1.4	0.007
Blood eosinophil ≥5%	22 (23.4)	2 (8.0)	0.088
Blood eosinophil ≥3%	47 (50.0)	7 (28.0)	0.050
Blood eosinophil,/μL (n = 119)	262.5 ± 237.0	164.5 ± 97.2	0.002
Blood eosinophil ≥500/μL	12 (12.8)	0	0.069
Serum IgE, U/mL (n = 68)	197.9 ± 468.0	215.9 ± 231.1	0.857
Serum IgE ≥100 U/mL	18 (30.5)	6 (66.7)	0.058
Positive skin prick test (n = 121)	17 (17.9)	4 (15.4)	1.000
Positive specific IgE to house dust mite (n = 62)	3 (5.4)	1 (16.7)	0.342
Postbronchodilator FEV_1_, % predicted	90.8 ± 22.5	98.2 ± 22.2	0.077
Persistent airflow limitation	90 (64.8)	16 (43.2)	0.018
Variable airflow limitation	72 (51.8)	15 (40.5)	0.224
Sputum eosinophil, %	12.5 ± 10.1	9.6 ± 6.6	0.040
Sputum neutrophil, %	2.5 ± 3.2	3.3 ± 11.8	0.679
Use of ICS during 1 year	100 (71.9)	25 (67.6)	0.602
MPR for ICS during 1 year, %	51.3 ± 38.9	34.4 ± 38.9	0.020
MPR for ICS ≥75%	50 (36.0)	6 (16.2)	0.022
MPR for ICS ≥50%	80 (57.6)	12 (32.4)	0.007

Data are given as mean ± SD or No. (%).

Abbreviations: ACT, asthma control test; CAT, COPD assessment test; FEV_1_, forced expiratory volume in 1 second; ICS, inhaled corticosteroid; MPR, medication possession ratio.

**Table 5:** Multivariable Analysis of Factors Associated With 1-year Improvement in Sputum Eosinophilia.

Variable	aOR^a^	95% CI	*P* Value
**1-year MPR for ICS** <**75%**			
Persistent airflow limitation: no (NAEB or asthma)	1		
Persistent airflow limitation: yes (COPD or possible ACOS^b^)	0.26	0.09–0.77	0.015
**1-year MPR for ICS** ≥**75%**			
Persistent airflow limitation: no (NAEB or asthma)	1		
Persistent airflow limitation: yes (COPD or possible ACOS^b^)	3.58	0.22–58.3	0.370

Abbreviations: ACOS, asthma-COPD overlap syndrome; aOR, adjusted odds ratio; CI, confidence interval; COPD, chronic obstructive pulmonary disease; ICS, inhaled corticosteroid; MPR, medication possession ratio; NAEB, nonasthmatic eosinophilic bronchitis.

^a^adjusted by blood eosinophil (≥5% vs <5%), serum IgE (≥100 U/mL vs <100 U/mL), FEV_1_ (% predicted), sputum eosinophil (%), and variable airflow limitation (yes vs no).

^b^When patients fulfilled both diagnostic criteria for asthma and COPD, we defined their condition as possible ACOS.

## Discussion

In summary, the incidence rate of acute exacerbations in NAEB was 0.13 per patient-year. We did not find that ICS therapy reduced the exacerbation rate in patients with NAEB. NAEB rarely progressed to chronic airflow obstruction. One fifth of patients with chronic airway diseases showed an improvement in eosinophilic airway inflammation at the 1-year follow-up. Patients with persistent airflow limitation (COPD or possible ACOS) showed a lower probability of improvement in sputum eosinophilia than those without persistent airflow limitation (NAEB or asthma).

Our study showed that NAEB patients did experience acute exacerbations during the follow-up period. As mentioned previously, NAEB patients could have respiratory symptoms other than cough, such as chest tightness with wheezing, shortness of breath, and sputum production^8–10^. Treatment with systemic corticosteroids is occasionally required to relieve these symptoms^11^. However, there were no studies investigating the incidence and predictors of acute exacerbations. Recently, the SPIROMICS cohort study—which used the same definition of an exacerbation—reported that symptomatic current or former smokers without COPD did experience exacerbations, and that their annualized exacerbation rate was significantly higher than those of asymptomatic current or former smokers and never-smokers (0.27, 0.08, and 0.03 events per year, respectively)^23^. The exacerbation rate of NAEB patients in our study was higher than that of asymptomatic smokers, but half that of symptomatic smokers in the SPIROMICS cohort who had preserved pulmonary function.

We did not find that ICSs prevented exacerbations in NAEB patients. In addition, the mean sputum eosinophil counts between baseline and the 1-year follow-up were not different regardless of ICS treatment (S3 Table and S1A Fig.). Little improvement in eosinophilic airway inflammation was in contrast to findings of previous prospective studies, in which all NAEB patients were treated with ICSs for at least 4 weeks^7,25^. The failure to show their effect on exacerbations and eosinophilic airway inflammation highlights that only a small number of patients have received adequate therapy in the real-world population of NAEB. In our study, only a quarter of NAEB patients were treated with ICSs for ≥50% of the follow-up days, and less than one sixth were treated with ICSs for ≥75% of the follow-up days. This might result in the lack of statistical power in the 1:1 PS matched analysis because only a small fraction of participants could be included in the analysis. Another potential cause of the real-world consequence in respect to lack of the efficacy of ICSs in preventing NAEB exacerbations is prevailing infectious triggers leading to aggravated symptoms. The definition of the exacerbation was non-discriminatory with respect to inherent worsening of eosinophilic airway inflammation versus infection by respiratory viruses or other infectious organisms. There may be a signal for a subset analysis where subjects with obvious infectious etiologies to exacerbations are excluded. However, due to the retrospective design of this study, we could not explicitly distinguish between exacerbations with and without infectious etiologies. Nevertheless, regardless of their effect on exacerbations and sputum eosinophils, ICSs played a role in improving symptoms in our study. The mean cough score had significantly reduced—from 2.3 to 1.6—at the 1-year follow-up in the eight NAEB patients whose MPR for ICSs was ≥50% (*P* = 0.049). In the 12 NAEB patients whose MPR for ICSs was <50%, the mean cough score had not changed significantly (*P* = 0.586; S3 Table and S1B Fig.).

We found that chronic airflow obstruction developed in relatively few NAEB patients, although it was limited by the relatively short follow-up period. According to previous studies by Berry *et al*.^5^ and Park *et al*.^6^, persistent airflow obstruction developed in approximately 15% of NAEB patients. However, in a recent investigation by Lai *et al*.^7^, none of the NAEB patients developed persistent airflow obstruction. This inconsistency regarding COPD development may have arisen because the studies had different proportions of smokers. Specifically, approximately 20% of NAEB patients were current or former smokers in the present study, and in that by Berry *et al*.^5^. In the study by Park *et al*.^6^, 46% of the participants were smokers. However, only 6% of NAEB patients were smokers in the study by Lai *et al*.^7^.

In a subgroup analysis, we showed that patients with persistent airflow limitation were less likely to show improvements in sputum eosinophilia. It has been reported that ICSs reduce the number of inflammatory cells in the bronchial mucosa and sputum^26^, and that the presence of eosinophilia in sputum^12,14^ and blood^27,28^ is a predictor of response to ICSs in COPD patients. However, no studies have compared the treatment response between COPD and other chronic airway diseases. In the present study, only 12% of patients with COPD showed an improvement in sputum eosinophilia, while more than a third of those with NAEB did. Corticosteroid resistance in COPD^29,30^ might explain the relatively poor treatment response to ICSs in our study.

The current study has several limitations, including its retrospective design of a cohort at a single institution. First, we could not standardize therapeutic plans of various chronic airway diseases, thus, therapies other than ICSs could affect airway eosinophilic inflammation. In addition, not all NAEB patients were evaluated with regard to whether their symptoms were improved by ICS therapy. Second, since we excluded patients with eosinophilic lung diseases based on chest radiographs, a possibility of systemic diseases such as vasculitis occurring without definite infiltration in their chest radiographs could not be excluded. Third, when determining the patients’ atopic status, not all patients underwent both skin prick testing and testing for the presence of specific IgE to house dust mites. However, over 80% of study patients in subcohort 1 & 2 underwent either skin prick testing to 55 common inhalant allergens or measurement of the specific IgE to house dust mites. Fourth, the main limitation regarding NAEB was the small number of NAEB patients followed up. Fifth, more symptomatic NAEB patients were likely to receive ICSs and adhere to them. To minimize this selection bias, we used PS matching to evaluate the effect of ICSs on the exacerbation rate. Finally, we did not apply the criteria recently suggested by investigators to diagnose ACOS^31–33^. However, we did analyze the improvement in sputum eosinophilia according to persistent and variable airflow limitation, rather than disease entities, in subcohort 2. For this reason, our definition of ACOS (dubbed “possible ACOS”) did not affect the validity of the analysis determining predictors of 1-year improvement in sputum eosinophilia.

In conclusion, exacerbations requiring systemic corticosteroids, antibiotics, or hospitalization did occur in NAEB patients, although infrequently. Among patients with chronic airway diseases, those with persistent airflow limitation were less likely to show improvement in eosinophilic airway inflammation.

## Electronic supplementary material

Supplementary information
