# Supplementary material for: Clinical Outcome of Eosinophilic Airway Inflammation in Chronic Airway Diseases Including Nonasthmatic Eosinophilic Bronchitis
Source: Sci Rep. 2018 Jan 9;8:146. doi: 10.1038/s41598-017-18265-2 (PMC5760521; doi:10.1038/s41598-017-18265-2)

# Clinical Outcome of Eosinophilic Airway Inflammation in Chronic Airway Diseases Including Nonasthmatic Eosinophilic Bronchitis

Jaeyoung Cho, Sun Mi Choi, Jinwoo Lee, Young Sik Park, Sang-Min Lee, Chul-Gyu Yoo, Young Whan Kim, Sung Koo Han, Chang-Hoon Lee

**S1 Table.** Baseline Characteristics of Patients With Nonasthmatic Eosinophilic Bronchitis Before and After Propensity Score Matching

| Characteristic | Before Propensity Score Matching | | | After Propensity Score Matching | | |
|---|---|---|---|---|---|---|
| | MPR for ICS < 50% (n = 113) | MPR for ICS ≥ 50% (n = 39) | *P* Value | MPR for ICS < 50% (n = 33) | MPR for ICS ≥ 50% (n = 33) | *P* Value |
| Age, y | 59.2 ± 14.0 | 58.1 ± 13.2 | .659 | 58.5 ± 13.3 | 59.2 ± 12.2 | .818 |
| Female sex | 79 (69.9) | 30 (76.9) | .402 | 25 (75.8) | 25 (75.8) | 1.000 |
| Smoking status | | | .882 | | | .714 |
| Never-smoker | 77 (68.1) | 29 (74.4) | | 29 (87.9) | 26 (78.8) | |
| Former smoker | 15 (13.3) | 3 (7.7) | | 2 (6.1) | 3 (9.1) | |
| Current smoker | 6 (5.3) | 2 (5.1) | | 1 (3.0) | 2 (6.1) | |
| Unknown | 15 (13.3) | 5 (12.8) | | 1 (3.0) | 2 (6.1) | |
| Baseline symptom scores (n = 103, n' = 46) | | | | | | |
| Cough score (n = 75, n' = 32) | 1.8 ± 1.3 | 2.5 ± 1.5 | .043 | 1.4 ± 1.3 | 2.3 ± 1.4 | .073 |
| CAT score (n = 76, n' = 33) | 11.9 ± 6.5 | 15.2 ± 5.9 | .054 | 11.6 ± 6.4 | 15.6 ± 6.3 | .082 |
| CAT score ≥ 10 | 34 (60.7) | 15 (75.0) | .252 | 9 (56.3) | 12 (70.6) | .392 |
| ACT score (n = 100, n' = 43) | 20.2 ± 4.2 | 17.4 ± 4.6 | .005 | 19.3 ± 4.7 | 18.0 ± 4.5 | .346 |
| ACT score ≥ 20 | 45 (63.4) | 13 (44.8) | .088 | 9 (45.0) | 12 (52.2) | .639 |
| White blood cell, /µL (n = 96, n' = 36) | 6073 ± 1647 | 6466 ± 2250 | .391 | 5855 ± 1385 | 6214 ± 2006 | .533 |
| Blood eosinophil, % (n = 96, n' = 36) | 2.8 ± 2.1 | 3.7 ± 2.7 | .140 | 3.5 ± 2.9 | 3.6 ± 2.8 | .950 |
| Blood eosinophil ≥ 5% | 10 (13.0) | 6 (31.6) | .081 | 6 (31.6) | 5 (29.4) | .888 |
| Blood eosinophil ≥ 3% | 29 (37.7) | 8 (42.1) | .722 | 8 (42.1) | 6 (35.3) | .676 |
| Blood eosinophil, /µL (n = 96, n' = 36) | 172.5 ± 131.8 | 248.2 ± 225.1 | .174 | 215.4 ± 198.9 | 227.1 ± 214.7 | .867 |
| Blood eosinophil ≥ 500/µL | 2 (2.6) | 3 (15.8) | .051 | 2 ( 10.5) | 2 (11.8) | 1.000 |
| Serum IgE, U/mL (n = 43, n' = 16) | 228.6 ± 758.2 | 94.2 ± 116.9 | .329 | 553.4 ± 1462.4 | 113.0 ± 127.9 | .394 |
| Serum IgE ≥ 100 U/mL | 10 (29.4) | 3 (33.3) | 1.000 | 2 (22.2) | 3 (42.9) | .596 |
| Positive skin prick test (n = 103, n' = 54) | 18 (25.4) | 4 (12.5) | .141 | 4 (14.3) | 4 (15.4) | 1.000 |
| Positive specific IgE to house dust mite (n = 30, n' = 12) | 1 (4.2) | 0 | 1.000 | 0 | 0 | – |
| Postbronchodilator $FEV_1$, % predicted | 109.4 ± 15.5 | 106.8 ± 21.4 | .495 | 111.0 ± 19.1 | 105.3 ± 22.3 | .272 |
| Postbronchodilator $FEV_1$/FVC, % | 80.3 ± 5.5 | 79.1 ± 6.2 | .266 | 79.5 ± 6.6 | 79.3 ± 6.5 | .925 |
| Bronchodilator response ($FEV_1$, %) | 3.3 ± 3.4 | 2.6 ± 3.6 | .252 | 3.0 ± 4.0 | 2.2 ± 3.5 | .358 |
| Bronchodilator response ($FEV_1$, mL) | 73.5 ± 80.3 | 62.8 ± 81.3 | .475 | 71.8 ± 94.7 | 53.9 ± 75.8 | .400 |
| Sputum eosinophil, % | 7.6 ± 5.8 | 12.0 ± 14.5 | .070 | 9.3 ± 5.8 | 9.5 ± 10.2 | .925 |
| Sputum neutrophil, % | 1.7 ± 2.6 | 1.6 ± 1.9 | .726 | 1.8 ± 1.8 | 1.6 ± 2.0 | .775 |

Data are given as mean ± SD or No. (%). The n is the number of patients before propensity score matching and n' is the number of patients after propensity score matching. Abbreviations: ACT, asthma control test; CAT, COPD assessment test; FEV1, forced expiratory volume in 1 second; FVC, forced vital capacity; ICS, inhaled corticosteroid; MPR, medication possession ratio.

**S2 Table.** Baseline Characteristics of Patients With Sputum Eosinophilia Before and After Propensity Score Matching

| Characteristic | Before Propensity Score Matching | | | After Propensity Score Matching | | |
|---|---|---|---|---|---|---|
| | 1-year MPR for ICS < 75% (n = 120) | 1-year MPR for ICS ≥ 75% (n = 56) | *P* Value | 1-year MPR for ICS < 75% (n = 47) | 1-year MPR for ICS ≥ 75% (n = 47) | *P* Value |
| Age, y | 65.8 ± 11.1 | 64.7 ± 12.8 | .558 | 64.4 ± 10.6 | 64.9 ± 11.7 | .840 |
| Female sex | 45 (37.5) | 21 (37.5) | 1.000 | 16 (34.0) | 17 (36.2) | .829 |
| Smoking status | | | .874 | | | .914 |
| Never-smoker | 49 (40.8) | 22 (39.3) | | 19 (40.4) | 17 (36.2) | |
| Former smoker | 49 (40.8) | 23 (41.1) | | 19 (40.4) | 21 (44.7) | |
| Current smoker | 20 (16.7) | 9 (16.1) | | 8 (17.0) | 7 (14.9) | |
| Unknown | 2 (1.7) | 2 (3.6) | | 1 (2.1) | 2 (4.3) | |
| Baseline symptom scores (n = 150, n' = 84) | | | | | | |
| Cough score (n = 98, n' = 48) | 1.7 ± 1.3 | 2.3 ± 1.4 | .046 | 2.1 ± 1.2 | 2.1 ± 1.5 | .916 |
| CAT score (n = 123, n' = 62) | 11.7 ± 7.5 | 16.6 ± 7.3 | .001 | 14.3 ± 7.1 | 16.0 ± 7.3 | .360 |
| CAT score ≥ 10 | 47 (55.3) | 33 (86.8) | .001 | 25 (83.3) | 27 (84.4) | 1.000 |
| ACT score (n = 137, n' = 80) | 20.1 ± 4.5 | 16.6 ± 5.1 | < .001 | 18.8 ± 4.9 | 16.7 ± 5.2 | .061 |
| ACT score ≥ 20 | 56 (60.9) | 17 (37.8) | .011 | 19 (46.3) | 15 (38.5) | .476 |
| White blood cell, /µL (n = 119, n' = 66) | 6697 ± 1946 | 7530 ± 2435 | .048 | 7285 ± 2427 | 7103 ± 2198 | .751 |
| Blood eosinophil, % (n = 119, n' = 66) | 3.2 ± 2.8 | 4.2 ± 3.0 | .085 | 3.8 ± 3.7 | 4.1 ± 3.0 | .683 |
| Blood eosinophil ≥ 5% | 12 (14.6) | 12 (32.4) | .025 | 8 (23.5) | 9 (28.1) | .670 |
| Blood eosinophil ≥ 3% | 35 (42.7) | 19 (51.4) | .379 | 15 (44.1) | 16 (50.0) | .632 |
| Blood eosinophil, /µL (n = 119, n' = 66) | 212.6 ± 201.2 | 306.8 ± 243.4 | .029 | 267.6 ± 269.9 | 282.1 ± 220.1 | .813 |
| Blood eosinophil ≥ 500/µL | 5 (6.1) | 7 (18.9) | .047 | 3 (8.8) | 5 (15.6) | .469 |
| Serum IgE, U/mL (n = 68, n' = 36) | 216.2 ± 508.1 | 164.8 ± 246.7 | .576 | 351.5 ± 784.8 | 184.3 ± 262.0 | .401 |
| Serum IgE ≥ 100 U/mL | 17 (36.2) | 7 (33.3) | .821 | 8 (44.4) | 7 (38.9) | .735 |
| Positive skin prick test (n = 121, n' = 71) | 13 (16.3) | 8 (19.5) | .654 | 4 (11.1) | 6 (17.1) | .514 |
| Positive specific IgE to house dust mite (n = 62, n' = 36) | 3 (7.1) | 1 (5.0) | 1.000 | 3 (16.7) | 1 (5.6) | .603 |
| Postbronchodilator $FEV_1$, % predicted | 92.0 ± 22.1 | 93.2 ± 23.7 | .743 | 92.3 ± 23.0 | 93.9 ± 23.5 | .740 |
| Postbronchodilator $FEV_1$/FVC, % | 65.1 ± 14.5 | 65.3 ± 13.4 | .925 | 65.6 ± 14.1 | 65.5 ± 12.6 | .971 |
| Bronchodilator response ($FEV_1$, %) | 7.5 ± 8.0 | 7.3 ± 9.9 | .911 | 8.9 ± 8.1 | 7.2 ± 10.6 | .372 |
| Bronchodilator response ($FEV_1$, mL) | 132.1 ± 119.1 | 131.1 ± 178.5 | .969 | 160.2 ± 123.2 | 132.1 ± 190.1 | .398 |
| Sputum eosinophil, % | 10.6 ± 7.8 | 14.8 ± 12.0 | .018 | 12.7 ± 8.6 | 12.3 ± 10.3 | .845 |
| Sputum neutrophil, % | 2.0 ± 2.0 | 4.0 ± 10.3 | .141 | 2.5 ± 2.6 | 2.2 ± 2.8 | .682 |

Data are given as mean ± SD or No. (%). The n is the number of patients before propensity score matching and n' is the number of patients after propensity score matching. Abbreviations: ACT, asthma control test; CAT, COPD assessment test; FEV1, forced expiratory volume in 1 second; FVC, forced vital capacity; ICS, inhaled corticosteroid; MPR, medication possession ratio.

**S3 Table.** The 1-year Changes of Sputum Eosinophils and Symptom Scores According to Medication Possession Ratio for Inhaled Corticosteroids

| Characteristic | 1-year MPR for ICS | Initial | After 1 year | *P* Value |
|---|---|---|---|---|
| Sputum eosinophil, % | < 50% (n = 25) | 7.2 ± 5.3 | 4.7 ± 6.4 | .189 |
| | ≥ 50% (n = 23) | 11.0 ± 12.5 | 9.4 ± 8.0 | .504 |
| Cough score | < 50% (n = 12) | 1.8 ± 1.0 | 1.9 ± 0.9 | .586 |
| | ≥ 50% (n = 8) | 2.3 ± 1.5 | 1.6 ± 1.2 | .049 |
| CAT score | < 50% (n = 12) | 11.8 ± 6.4 | 12.4 ± 7.7 | .707 |
| | ≥ 50% (n = 8) | 14.8 ± 8.3 | 15.3 ± 9.8 | .747 |
| ACT score | < 50% (n = 13) | 21.7 ± 3.2 | 21.7 ± 4.0 | 1.000 |
| | ≥ 50% (n = 14) | 18.9 ± 5.0 | 21.2 ± 3.3 | .091 |

Data are given as mean ± SD.

Abbreviations: ACT, asthma control test; CAT, COPD assessment test; ICS, inhaled corticosteroid; MPR, medication possession ratio.

**S1 Fig.** The 1-year changes in sputum eosinophils (A) and the cough score (B) according to medication possession ratio for inhaled corticosteroids. ICS, inhaled corticosteroid; MPR, medication possession ratio

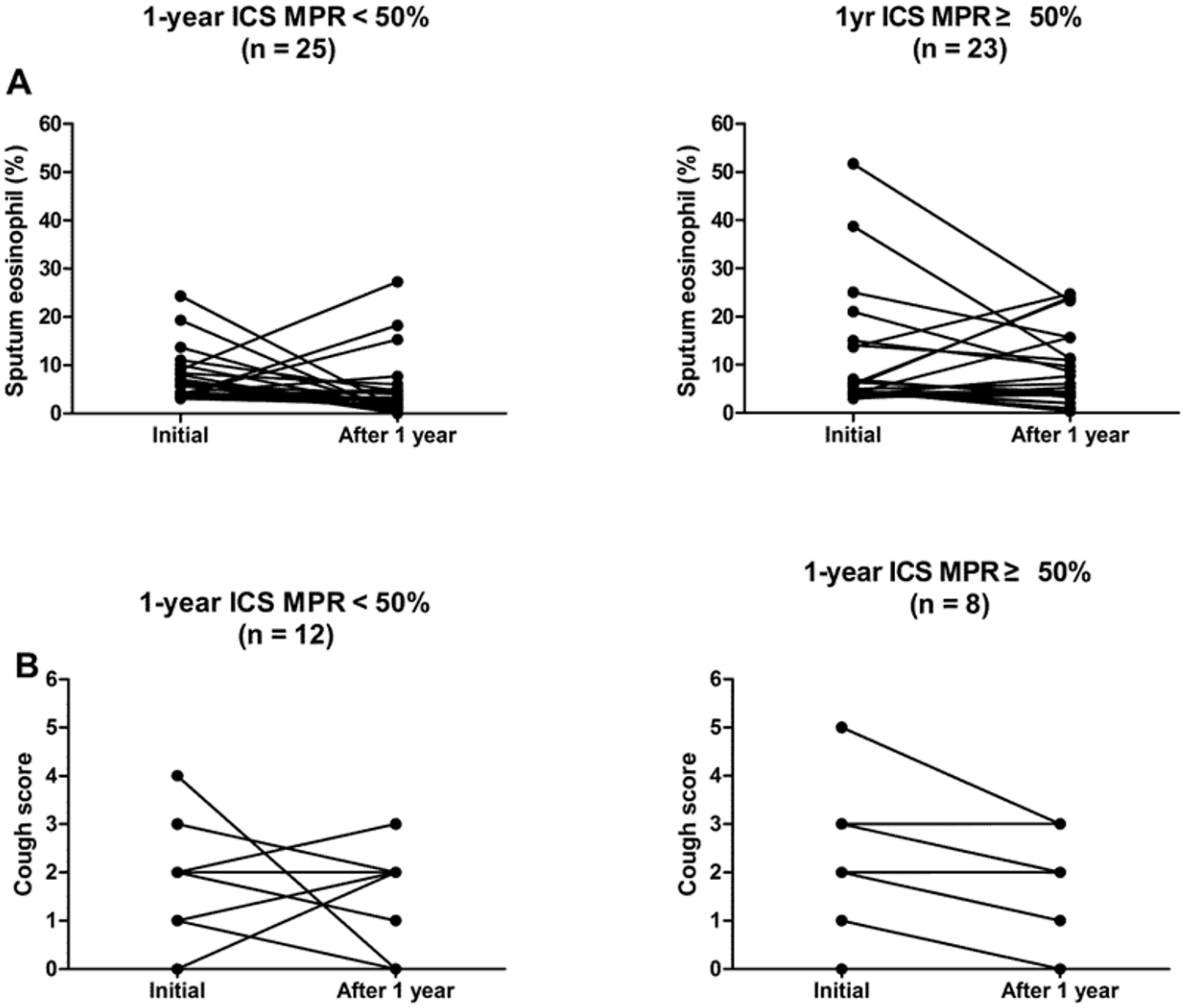

Supplement: Supplementary file 1 — Supplementary information [file 41598_2017_18265_MOESM1_ESM.pdf]
